# The relationship between C-reactive protein and prognostic factors in chronic obstructive pulmonary disease

**DOI:** 10.1186/2049-6958-8-63

**Published:** 2013-09-28

**Authors:** Reshu Agarwal, Mohammad Shoaib Zaheer, Zubair Ahmad, Jamal Akhtar

**Affiliations:** 1 Department of Medicine, Jawaharlal Nehru Medical College, Aligarh Muslim University, Aligarh 202002, India; 2 Department of Tuberculosis & Respiratory Diseases, Jawaharlal Nehru Medical College, Aligarh Muslim University, Aligarh 202002, India

**Keywords:** High sensitivity-C-reactive protein, Chronic obstructive pulmonary disease

## Abstract

**Background:**

The purpose of the study was to determine the relationship between high sensitivity C-reactive protein (hs-CRP) levels and prognostic factors in chronic obstructive pulmonary disease.

**Methods:**

We studied 50 stable COPD patients with: spirometry, 6 minute walk distance, body mass index, GOLD stage (spirometric classification) and smoking status. In these patients hs-CRP values were measured and compared with those of 50 healthy controls. Then the serum hs-CRP was subjected to evaluation for any correlation with the predictors of outcomes in COPD subjects.

**Results:**

Hs-CRP levels were higher in COPD patients than in controls (4.82 vs. 0.88 mg/L p < 0.01). Correlation was found between hs-CRP and the following variables: FEV_1_ (r= −0.813; p < 0.01), 6MWD (r= −0.876; p < 0.01), body mass index (r= −0.710; p < 0.01), GOLD stage (r= 0.797, p < 0.01) and smoking status (r= 0.796; p < 0.01). Using multivariate analysis, FEV_1_ and 6MWD showed the strongest negative association with hs-CRP levels.

**Conclusions:**

The circulating levels of the inflammatory marker hs-CRP are significantly elevated in patients with COPD, supporting the view that COPD is in part an inflammatory disorder. Hs-CRP levels in stable COPD patients are best correlated with FEV_1_ and 6-minute walk distance (6MWD). This information should be considered when hs-CRP levels are measured in stable COPD patients.

## Background

Chronic obstructive pulmonary disease (COPD) is a syndrome characterised and defined by a single physiological parameter: limitation of expiratory air-flow which, most often, is slowly progressive over the years [1]. According to the widely accepted definition from Global Initiative on Obstructive Lung Disease (GOLD) COPD is "a disease state characterized by airflow limitation that is not fully reversible. The airflow limitation is usually progressive and associated with an abnormal inflammatory response of the lungs to noxious particles and gases" [2]. For the first time this definition encompasses the idea that COPD is a chronic inflammatory disease and much of the recent research has focused on the nature of this inflammatory response. Numerous studies performed in recent years provide overwhelming evidence of COPD as a condition characterized by an abnormal inflammatory response beyond the lungs with evidence of low-grade systemic inflammation which causes systemic manifestations such as weight loss, skeletal muscle dysfunction, an increased risk of cardiovascular disease, osteoporosis and depression, among others [3-6]. Blood markers, such as IL-6, CRP and fibrinogen have attracted interest during recent years, and further studies in this area will probably increase the understanding of systemic manifestations in COPD [7].

One of the inflammatory markers which is increasingly evaluated in COPD patients is C-reactive protein (CRP) [8]. C-Reactive Protein (CRP) is an acute phase protein synthesized predominantly by the hepatocytes in response to tissue damage or inflammation. It has been accepted that levels of CRP relate to the presence of airflow obstruction [6]. There are various studies evaluating the relationship between CRP levels and other clinical variables known to predict outcome in patients with COPD.

The present study aims to evaluate the levels of high sensitivity CRP (hs-CRP) in patients with COPD and to study the relationship between hs-CRP levels and prognostic factors in COPD patients.

## Methods

The present study was conducted at the Department of Medicine, JN Medical College Hospital, AMU, Aligarh from January 2008 to June 2009. The permission for the study was taken from ethical committee. A total of 100 subjects, including 50 control and 50 patients of chronic obstructive pulmonary disease (COPD), were recruited in the study.

Patients who had dyspnea, chronic cough, sputum production and risk factors, such as tobacco use and occupational exposures to dust and chemicals with all degree of airflow severity were consecutively included if they had a postbronchodilator FEV_1_/FVC of <0.7 after 400 micrograms of inhaled salbutamol. Patients were clinically stable (no exacerbation for 2 months) at the time of evaluation. An informed consent was obtained from each subject prior to entering the study.

Patients were excluded if they had a history of asthma and/or the FEV_1_ increased more than 12% or 200 ml following inhalation of 400 μg salbutamol, if they had acute coronary syndromes, collagen vascular/autoimmune diseases, malignancy, pulmonary embolism, renal insufficiency, cirrhosis and other serious liver diseases and if they were on steroid treatment.

A detailed history and physical examination was carried out for every subject who entered in the study as per a pre-designed proforma, including thorough physical examination, assessment of vital parameters, anthropometry (height, weight, BMI) and systemic examination for assessing the signs of COPD and also the presence of any exclusion criteria as previously discussed.

We evaluated the following variables known to predict outcome in COPD: degree of airflow obstruction by FEV1, exercise capacity by the six minute walk distance (6MWD) and body mass index (BMI). We also recorded if patients were active smokers or quitters (those who had left smoking ≥1 year).

BMI was calculated as the weight in kilograms divided by height in meters^2^. The hs-CRP estimation was performed by using UBI MAGIWEL CRP-quantitative AD-401 kit, a solid phase enzyme linked immunosorbent assay (ELISA) as per instructions of the manufacturer (supplied with kit) with reference range for CRP being 0.0-0.8 mg/dl. All patients underwent physiologic evaluation that included measurement of the forced expiratory volume in 1-second (FEV_1_) and forced vital capacity (FVC) using a spirometer (P. K. Morgan LTD, Kent, England) with published predicted values. Quality control and procedures of lung function testing were performed according to the European Respiratory Society guidelines. The 6MWD test carried out in a corridor 50 m long. Statistical analysis was performed using SPSS version 10.0 Statistical package for Windows (SPSS, Chicago, IL). Continuous variables were expressed as mean ± standard deviation (Gaussian distribution) or range. Unpaired and paired t tests for independent and dependent samples were used to compare continuous data between two groups. ANOVA or analysis of variance with Scheffe’s *post hoc* analysis were used for comparing data between groups. Linear relationship between variables was analyzed using Pearson’s correlation coefficient and significance of ‘r’ was tested. A Stepwise multivariate regression analysis was used to study the determinants of CRP. All p values were two tailed and values of < 0.05 were considered to indicate statistical significance. All confidence intervals were calculated at 95% level.

### Results

The study recruited 50 patients of with COPD (44 males and 6 females). We also recruited 50 healthy, non smoking subjects (44 males, 6 females) from the general population of similar age and location. Most of patients were in GOLD stages II and III and 76% of them were still smoking. The clinical and physiologic characteristics of the COPD and control group are presented in Table 1. Distribution of subjects according to GOLD stage(spirometric classification) and smoking status is shown in Tables 2 and 3.

**Table 1:** Clinical and physiologic characteristics of the COPD and control group

	**Control group**	**COPD group**	**P**
**Age(yrs)**	53.65 ± 8.68	54.00 ± 7.58	0.**628**
**M**:**F ratio**	**44**:**6**	**44**:**6**	0.**546**
**BMI(kg**/**m**^**2**^**)**	**22**.**55** ± **1**.**98**	**22**.**82** ± **2**.**63**	0.**679**
**FEV**_**1**_**(%)**	**88** ± **3**.**46**	**57**.**54** ± **18**.**33**	0.**000**
**6MWD(mtrs)**	**375**.**45** ± **68**.**98**	**212**.**60** ±**53**.**22**	0.**000**

**Table 2:** **Distribution of subjects according to GOLD stage**(**spirometric classification**)

**GOLD stage**	**No. of subjects**	**Percentage (%)**
**Stage I**	6	12
**Stage II**	22	44
**Stage III**	17	34
**Stage IV**	5	10

**Table 3:** Distribution of subjects according to smoking status

**Smoking status**	**No**. **of subjects**	**Percentage (%)**
**Smokers**	38	76
**Quitters**	12	24
**Total**	50	100

**Figure 1:**
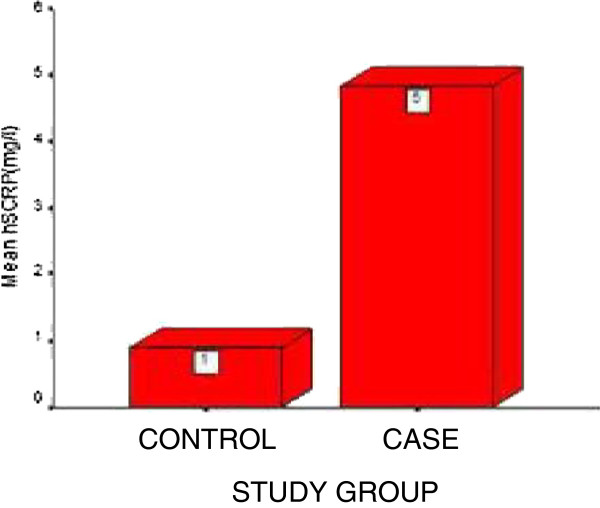
**Showing CRP levels in COPD and control subjects**.

Mean value of hs-CRP in control group was 0.88 ± 0.48 mg/l (range: 0.2-1.8 mg/l) while 4.82 ± 1.97 mg/l (range: 1.7-8.1 mg/l) in COPD group. When compared with controls by independent samples t-test, the mean value of hs-CRP was significantly higher in patients with COPD (11.574, p < 0.01) (Figure 1).

Pearson’s correlation coefficient showed that (Table 4) hs-CRP levels significantly correlated with GOLD stage (r = 0.797; p < 0.01). A significant negative correlation of hs-CRP was found with body mass index (r = −0.710; p < 0.01), FEV_1_ (r = −0.813; p < 0.01) and 6MWD (r = −0.876; p < 0.01). hs-CRP was found to be significantly higher in smokers as compared to quitters (r = 0.796; p < 0.01). There was no correlation of hs-CRP with sex. Spearman^’^s correlation was used to find out the correlation of hs-CRP with sex, smoking status and GOLD stage.

**Table 4:** **Pearson**’**s correlation of hs**-**CRP with other variables**

	**Correlation coefficient(r)**	**P Sig. (2-tailed)**
Hs-CRP(mg/l)	1.000	0.
Smoking status	.796	0.000
Sex	-.019	0.877
BMI(kg/m^2^)	-.710	0.000
FEV_1_ (%)	-.813	0.000
6MWD(m)	-.876	0.000
GOLD stage	.797	0.000

### Multistep linear regression analysis using hs-CRP as dependent variable in COPD subjects

Multistep linear regression analysis was done on 50 subjects using hs-CRP as the dependent variable and age, BMI, FEV_1_, 6MWT, smoking status and GOLD stage as covariates. The statistical details of the model are shown in Table 5.

**Table 5:** Multistep linear regression model

**Model**	**Variables**	**R**	**R**^**2**^	**β**	**T**	**P**
**1**	**6MWT**	0.888	0.789	- 0.888	- 15.937	0.000
**2**	**6MWT**	0.934	0.873	- 0.497	- 6.805	0.000
	FEV_1_			−0.487	−6.662	0.000

Stepwise multiple regression analysis was conducted to find out significant predictors of hs-CRP. 6MWT and FEV_1_ entered hierarchically in the regression model as significant predictors of hs-CRP.

## Discussion

Our study showed that the circulating levels of hs-CRP were significantly elevated in patients with COPD compared with controls (4.8 vs. 0.8 mg/l) (t=11.574, p < 0.01). The main finding of our study is that CRP level in stable COPD patients has the strongest association with 6MWD and FEV_1_. We also found that CRP levels correlated independently with other important prognostic clinical variables: namely BMI and GOLD stage(spirometric classification). Some studies have demonstrated elevated levels of CRP and fibrinogen in patients with COPD [9,10] and a meta-analysis by Gan et al. confirmed a significant increase in CRP levels in COPD patients compared with controls indicating a persistent systemic inflammation in subjects with COPD [4]. Yende et al. reported a higher level of serum CRP in cases with an obstructive pattern in their spirometry (3.5 mg/l) in comparison to normal population (2.5 mg/l) (p < 0.0001) [8]. In a study conducted by Broekhuizen et al., stable COPD patients had increased levels of inflammatory markers like CRP [11]. F. Karadag, found out that serum CRP was significantly higher in stable COPD patients than in control subjects (p < 0.001) [12]. This study confirms that circulating CRP levels are higher in stable COPD patients and may thus be regarded as a valid biomarker of low-grade systemic inflammation.

In our study, hs-CRP was found to be significantly higher in smokers as compared to quitters (r = 0.796; p < 0.01). Our work duplicates the previous finding of Juan P de Torres et al. regarding higher CRP levels in those COPD patients who are active smokers compared with those who are not [13,14]. Unfortunately, this effect could not be confirmed in the control group because we only recruited non-smoking individuals to represent the “normal” population. However, the level of CRP in the ex-smoking COPD population remained significantly higher than in the non-smoking control group. In contrast to the above findings, Pinto-Plata et al., showed a significantly higher level of CRP in COPD patients (50.03 ± 1.51 mg/l) than in smoking (2.02 ± 1.04 mg/l) and non smoking control groups (2.24 ± 1.04 mg/l) (p < 0.001) [6].

The authors suggest that, although cigarette smoking has a role in promoting inflammatory process in COPD patients, it is not the leading cause of increased inflammatory markers. It should be noticed that only some cases develop inflammatory reaction following cigarette smoking, and this can be due to genetic differences.

In our study, CRP is inversely correlated with FEV_1_. In assessing the association between hs- CRP and lung function, Shaaban et al., looked at cross-sectional and longitudinal changes between CRP and FEV_1_ decline [15]. Their analysis included 531 subjects demonstrating a negative association between FEV_1_ and CRP(p = 0.002) and higher CRPlevels over time were associated with a faster FEV_1_ decline. Similarly, a recent study found CRP levels associated with accelerated decline in FEV_1_ and mortality in patients with mild to moderate COPD, indicating that CRP measurements might enable identification of patients at a high risk of disease progression and mortality [16].

We observed that CRP levels are inversely correlated with 6MWD. Koechlin et al., found that CRP levels were inversely correlated with endurance time and Broekhuizen et al., also found that CRP increases in those patients with poor exercise capacity [11,17]. The study by Pinto Plata and colleagues takes these findings one step further. They evaluated 88 patients with COPD and 71 controls and subjected them to extensive physiological testing and a detailed review of medications. Consistent with the results reported by Broekhuizen et al. they found that serum CRP levels were inversely related to the distanced achieved in the 6MWD independent of other factors such as age, sex and smoking history [6].

One of the important extrapulmonary manifestations of COPD is skeletal muscle dysfunction and wasting [18]. With increasing severity of disease, patients with COPD lose muscle bulk, especially in their thighs and upper arms. Over time, these patients lose exercise endurance and complain of fatigue and dyspnea with only a minimal degree of exertion [19]. These symptoms curtail their ability to exercise and compromise their cardiac fitness, which further limits their exercise tolerance, creating a vicious downward spiral that can eventually lead to generalized debility and immobility [20]. Indeed, some authors postulate that the skeletal muscle dysfunction is a direct consequence of the systemic effects of the disease [21] whereas others propose that the “myopathy” is an independent process that contributes to the systemic inflammatory load of the disease [22]. Whatever the mechanism, our results indicate that measuring CRP levels in stable conditions could indirectly reflect the exercise capacity of these patients, an important prognostic factor of the disease. Encouragingly, early interventions with exercise programmes may restore some of the lost health status related to muscle dysfunction and increase patients' exercise tolerance and stamina. Collectively, these studies have extended our concept of COPD beyond the pulmonary system, provided a solid clinical and epidemiological rationale for linking systemic inflammation with peripheral muscle dysfunction and raised the possibility of using anti-inflammatory treatment to mitigate systemic inflammation in the hope of improving health outcomes in these patients.

Interestingly, in our study, BMI is inversely correlated inversely with CRP. This contrasts with the study by Marie-Kathrin Breyer et al., who found that obese COPD patients (BMI ≥ 30 kg/m^2^) were 3.3 times more likely (95% CI, 1.5-7.0, *p* = 0.002) to have highly elevated CRP levels compared to normal weight (BMI 21-24.9 kg/m^2^) COPD patients, after taking clinically relevant confounders into account [23]. In contrast, COPD patients with a low BMI (<21 kg/m^2^) were 2 times less likely (OR, 0.5; 95% CI, 0.3-0.9, *p* = 0.022) to have highly elevated CRP levels compared to normal-weight peers. Schols et al., observed high CRP level in a special subset of 16 COPD patients with high resting energy expenditure (REE) and low fat free mass (FFM) [24]. More studies are needed to help resolve these controversial findings. It has been proposed that inflammatory cytokines could be secreted by adipocytes and by inflammatory cells present in adipose tissue [25]. Further research is needed to elucidate the effect of different cytokines on body composition and vice versa.

Regarding the severity of disease based on GOLD criteria (spirometric classification), the mean serum CRP level was found to be significantly increased in severe cases. Pinto-Plata et al. [6] showed that there was no significant difference between the severity of disease and serum CRP level but de Torres and co-workers [13] indicated that serum CRP level significantly increased with the aggravation of disease. Therefore, although we expect the inflammatory process to be worse and the inflammatory markers to be increased by increasing the severity of disease, more studies are required in this regard.

## Conclusions

In summary, our study confirms that C-reactive protein levels are increased in stable chronic obstructive pulmonary disease patients. The CRP levels are associated with important clinical variables that help in predicting outcome of the patients. Among them, the most important are FEV_1_ and 6MWD. This finding reinforces and supports the relevance of CRP measurement and we suggest that the information here presented should be considered when CRP levels are measured in stable COPD patients. Further follow up cohort studies with greater samples and measuring CRP levels prospectively should help to determine the validity of our findings.

## Competing interests

The authors declared that they have no competing interests.
